# The association between glaucoma and all-cause mortality in middle-aged and elderly Chinese people: results from the China Health and Retirement Longitudinal Study

**DOI:** 10.4178/epih.e2023066

**Published:** 2023-07-21

**Authors:** Xiaoxu Huang, Mengqiao Xu, Minwen Zhou, Wenjia Liu, Xiaohuan Zhao, Xiaodong Sun

**Affiliations:** 1 Department of Ophthalmology, Shanghai General Hospital (Shanghai First People’s Hospital), Shanghai Jiao Tong University School of Medicine, Shanghai, China; 2 National Clinical Research Center for Eye Disease, Shanghai, China; 3 Shanghai Key Laboratory of Ocular Fundus Diseases, Shanghai, China

**Keywords:** Glaucoma, Mortality, China Health and Retirement Longitudinal Study, Risk factors

## Abstract

**OBJECTIVES:**

This population-based, prospective cohort study investigated the association between glaucoma and mortality in older adults.

**METHODS:**

Participants aged 45 years or older at baseline (47.9% male) were enrolled in 2011 for the China Health and Retirement Longitudinal Study (CHARLS). All-cause mortality was observed during 7 years of follow-up. The baseline data were collected in the 2011 CHARLS, and participants were followed up for 7 years (until 2018). The risk of all-cause mortality was investigated using Cox proportional-hazards regression with age as the time scale, adjusting for significant risk factors and comorbid conditions.

**RESULTS:**

Among the 14,803 participants included, the risk of all-cause death was significantly higher among people with glaucoma than among those without glaucoma, after adjustment for other confounders (hazard ratio [HR], 1.46; 95% confidence interval [CI], 1.04 to 2.03). In a subgroup analysis based on the mean age of death, among those who were 75 years and older (n=1,231), the risk of all-cause death was significantly higher in patients with glaucoma than in those without glaucoma (HR, 1.89; 95% CI, 1.24 to 1.89).

**CONCLUSIONS:**

Participants with glaucoma had a higher risk of all-cause mortality, especially those aged 75 years and above. Our findings revealed potential mechanisms underlying an association between glaucoma and all-cause mortality. They also highlighted the importance of glaucoma management to prevent premature death in middle-aged and older adults.

## INTRODUCTION

Glaucoma is a chronic, progressive disease characterised by structural changes to the optic nerve head and visual field loss [1]. Worldwide, the number of glaucoma patients has increased rapidly in recent decades, and it is expected to reach 112 million by 2040 [2]. In China, 13.12 million patients had glaucoma in 2015, accounting for 2.58% of the total population. The prevalence is estimated to increase to 3.48% by 2050, when 25.16 million people will be diagnosed with glaucoma in China [3]. Glaucoma has an uncertain prognosis and requires lifelong management and follow-up to prevent further loss of vision [4]. Therefore, it poses a major public health challenge with considerable social and economic impacts in China, which has one of the highest totals of glaucoma cases worldwide [5,6]. As the most frequent cause of blindness [7,8], glaucoma has a serious impact on life expectancy, especially for the elderly. A possible association between glaucoma and mortality has been noted since 1950 [9].

The relationship between glaucoma and mortality varies significantly in different studies. The Beijing Eye Study, using a multivariate model, observed a higher mortality rate in angle-closure glaucoma patients [10]. Furthermore, studies analysing Taiwanese health insurance data and an Indian rural cohort reported that primary open-angle glaucoma (POAG) was associated with higher 10-year mortality [11,12]. However, a meta-analysis of 9 studies presented no convincing evidence for a higher risk of all-cause mortality among those with POAG [13]. Similarly, there was no evidence of an association between POAG and survival in a Scandinavian cohort that was followed for up to 30 years [14]. Interestingly, in the population-based National Health and Nutrition Examination Survey (NHANES), a representative health survey conducted in the United States, glaucoma was related to a higher incidence of mortality in an unadjusted Cox regression model, but the correlation disappeared when the model was adjusted for age and sex [15].

In view of the anticipated increase in the incidence of glaucoma worldwide and the uncertainty of the evidence concerning the associated risk of death, there is a need for further research into the life expectancy of glaucoma patients [13,16]. Therefore, our research evaluated the relationship between glaucoma and mortality through the population-based China Health and Retirement Longitudinal Study (CHARLS). We investigated whether glaucoma is associated with increased mortality in subjects affected by comorbidities (such as diabetes or hypertension) and considered other potentially confounding factors.

## MATERIALS AND METHODS

### Study population and design

The CHARLS is a nationally representative longitudinal survey of middle-aged and elderly Chinese people (45 years and older). The national public database covers a wide range of information concerning family, health status and function, health care and insurance, work, retirement and pension, and physical measurements. A multistage probability sampling method was applied through the CHARLS, which covered 28 provinces, 150 counties and districts, and 450 villages or urban communities across China. A total of 17,705 individuals were recruited between June 2011 and March 2012 [17]. All participants underwent face-toface, computer-assisted personal interviews, which included a structured questionnaire, and follow-up occurred every 2 years. Physical measurements, including height, weight, and blood pressure, were evaluated at each follow-up, and blood samples were collected once in every 2 follow-up cycles. CHARLS has been described in more detail elsewhere [18].

### Assessment of glaucoma

Glaucoma was assessed via self-reports both at baseline and during follow-up. The presence of glaucoma was defined by a selfreported answer to the question, “Has a doctor, nurse, or paramedical ever treated you for glaucoma?” If the respondent answered “yes” to this question, the conclusion was that they had glaucoma. Participants with a pre-existing glaucoma diagnosis at baseline were excluded from the present study, and the enrolled participants received follow-up glaucoma assessments in 2013, 2015, and 2018. The range of glaucoma considered includes all types: POAG, primary angle-closure glaucoma, and secondary glaucoma.

### Follow-up of all-cause mortality

Participants enrolled in wave 1 (2011, baseline) were followed up in waves 2, 3, and 4 (2013, 2015, and 2018). Interview status (dead or alive) and date of death were recorded in wave 4. For those who had died from any cause, the survival time was calculated as the interval between baseline and the date of death. If the exact date of death was not available, the survival time was defined as the median of the interval between the baseline time and the specific wave in which information about the death was received. For those who had not died during the follow-up period, their survival time was considered to be the interval between 2 interview waves.

### Confounding variables

Information that included age, sex, educational level (primary or below, middle school, high school, or college and above), marital status (married or partnered, or otherwise), smoking (yes or no), and drinking (drinking more than once a month, drinking less than once a month, or none) was collected by trained interviewers using the 2011 CHARLS questionnaire. Respondents were categorised into different groups. Body mass index (BMI) was used to describe the obesity status of respondents and was defined as one’s body mass divided by the square of their height in units of kg/m2. Hypertension was defined as blood pressure ≥ 140/90 mmHg at baseline or a self-reported history of antihypertensive medication use. Dyslipidaemia was defined by having a self-reported dyslipidaemia history or as having more than one of the following: total cholesterol ≥ 6.2 mmol/L, low-density lipoprotein cholesterol ≥ 4.1 mmol/L, triglycerides ≥ 2.3 mmol/L, or high-density lipoprotein cholesterol < 1.0 mmol/L. Diabetes was defined as a selfreported diagnosis of diabetes, as a fasting plasma glucose level ≥ 7.0 mmol/L, or as glycated haemoglobin 6.5% [19].

### Serum measurements

Trained nurses collected 8 mL of fasting blood samples at township health centres or at the local offices of the Chinese Centre for Disease Control and Prevention (CDC) after participants had fasted for at least eight hours overnight. The whole blood and centrifugal serum were transported to the CDC at -20°C and stored at -80°C. Levels of plasma glucose, lipids, and glycosylated haemoglobin were then measured in a laboratory at Capital Medical University.

### Statistical analysis

Continuous data were expressed as mean± standard deviation and analysed using the Student t-test for group comparisons, whereas categorical data were presented as numbers (n) with percentages (%) and analysed using the chi-square test for group comparisons. The overall significance of the univariate survival analysis was determined by the log-rank test using the Kaplan-Meier analysis. Cox proportional-hazards regression analyses were carried out according to glaucoma status to obtain hazard ratios (HR) and 95% confidence intervals (CIs) for risk factors significantly associated with mortality. The proportional-hazard assumption was tested based on Schoenfeld residuals and the Kaplan-Meier survival curve, and no violation was found. The Cox proportional-hazards regression model was adjusted for potentially confounding factors. A subgroup analysis was conducted based on the average age at death, and participants were divided into 2 subgroups: those aged 45-74 years and those 75 years and older. All statistical analyses were conducted using SAS version 9.4 (SAS Institute Inc., Cary, NC, USA), and a 2-sided p-value < 0.05 was considered statistically significant.

### Ethics statement

CHARLS was approved by the Ethical Review Committee at Peking University.

## RESULTS

**Figure 1.**
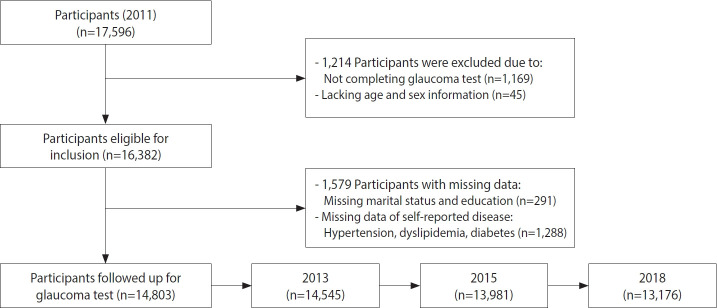
Flow chart of the study sample of middle-aged and older Chinese adults: China Health and Retirement Longitudinal Study, 2011- 2018.

In total, 14,803 participants were included for analysis after filtering (Figure 1). Table 1 shows the baseline characteristics of the participants in our study. Of the 14,803 participants meeting the criteria for enrolment, 141 (0.9%) were diagnosed with glaucoma. Compared to the participants with glaucoma, those without glaucoma were younger (66.6 ± 10.1 vs. 59.0 ± 9.9, p < 0.001), were more likely to be male, educated, and married or partnered; to smoke; and to consume alcohol; and less likely to have hypertension, dyslipidaemia, or diabetes.

**Figure 2.**
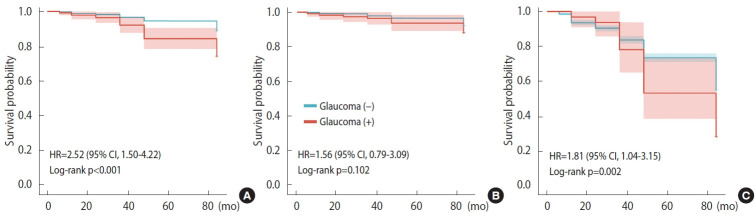
Data on the survival probability of individuals with glaucoma (A) all ages, (B) aged 45-74 years, and (C) aged 75 years and older. HR, hazard ratio; CI, confidence interval.

Figure 2 presents the survival probability of the groups with and without glaucoma by Kaplan-Meier analysis. In general, across all age-based strata, the survival probability of individuals diagnosed with glaucoma was significantly lower than that of the non-glaucoma group (p< 0.001, Figure 2A). We further stratified patients by average age of death and found that survival probability was significantly associated with glaucoma status in people aged 75 years and older (p= 0.002, Figure 2C), but not in people aged 45-74 years (p= 0.102, Figure 2B).

During the 7-year follow-up, there were 1,627 deaths among all participants. At baseline, the participants who died with glaucoma were significantly older than those who died without glaucoma (75.94 vs. 68.99 years, p< 0.001), were less likely to be male (38.9 vs. 58.9%, p= 0.016), and were less likely to smoke (30.6 vs. 51.0%, p= 0.015) (Supplementary Material 1). Considering the strong association between age and death over time, we further stratified by age according to average age at death to explore whether glaucoma status was associated with all-cause mortality. The characteristics of patients who died according to glaucoma status, stratified by age group, are shown in Supplementary Material 2. Among individuals between 45 years and 74 years at time of death, the age distribution differed between the glaucoma and non-glaucoma groups.

Risk factors associated with all-cause mortality are presented in Tables 2 and 3. The multivariate Cox regression analysis indicated that individuals with glaucoma had a higher risk of all-cause mortality than those without glaucoma (HR, 1.46; 95% CI, 1.04 to 2.03), even if adjusting for age, sex, and comorbid conditions. In addition, lifestyle factors, such as smoking and drinking, and chronic systemic diseases, including hypertension and diabetes, increased the risk of all-cause mortality. Subjects with a low educational level also had a higher risk of all-cause mortality. Being married or living with a partner correlated to a lower risk of allcause mortality. In the Cox proportional-hazards regression analysis, elevated BMI was not associated with excess all-cause mortality risk. A higher BMI was even found to be a protective factor, with statistical significance (HR, 0.92; 95% CI, 0.91 to 0.94).

After being stratified by age, individuals 75 years and older with glaucoma still showed a higher risk of all-cause mortality after age and concomitant conditions were considered (HR, 1.89; 95% CI, 1.24 to 2.89; Table 3). The predictive factors for mortality among such individuals included having less education, never having married or had a partner, smoking, and having hypertension. In the younger group, the factors predictive of mortality included being male, having less education, never having married or had a partner, smoking, drinking, having hypertension, and having diabetes.

## DISCUSSION

Overall, our current results based on a set of representative Chinese people strongly suggest that glaucoma is a high-risk factor for all-cause mortality in the middle-aged and elderly population, especially among people older than 75 years. In our study, among participants of all ages, the presence of glaucoma is accompanied by a higher risk of all-cause mortality (HR, 1.46; 95% CI, 1.04 to 2.03), even after considering other conditions that might affect the risk of mortality, such as age, sex, BMI, smoking, drinking, hypertension, dyslipidaemia, and diabetes. When stratified by mean age of death, glaucoma significantly increases all-cause mortality in individuals in their mid-70s and older (HR, 1.89; 95% CI, 1.24 to 2.89). However, among younger individuals of 45 years to 74 years of age, those with glaucoma are not at higher risk compared with their same-age peers.

The results of our study agree with many previous findings [10,11,20-25]. The National Health Interview Survey (NHIS) [25] reported that the probability of death from any cause occurring over a median of 7 years of follow-up was higher (HR, 1.35; 95% CI, 1.19 to 1.53) among participants with glaucoma compared to those without glaucoma, even after adjustment for confounders. The NHIS survey also reported a higher risk of mortality from cardiovascular disease among participants with glaucoma. Similarly, a Taiwanese study [11] demonstrated higher mortality associated with POAG (adjusted HR, 2.11; 95% CI, 1.76 to 2.54), and it observed an association between glaucoma and a higher risk of acute renal failure. Moreover, the Beijing study, which examined 4,356 subjects for glaucoma, suggested that glaucoma, especially angle-closure glaucoma, might be associated with an increased rate of mortality among Chinese adults in Greater Beijing [10]. Previous studies on the relationship between glaucoma and all-cause mortality were mainly conducted in developed countries, such as Australia, the United States, and Sweden [25-27]. In the following decade, relevant studies on all-cause mortality of glaucoma were also carried out in some areas of China. Few studies focused on glaucoma patients in the whole country, however, and most of them were small-scale studies, limited to local sampling in individual provinces and cities, such as studies conducted in Taiwan study [11] and Beijing [10]. The sampling coverage of our study was broader. Due to China’s vast territory and large population, people in different regions also show different characteristics of eye diseases. For example, the incidence of glaucoma in northern China, such as Inner Mongolia and Harbin, is 1.4% and 0.7%, respectively [28,29], while the incidence of glaucoma in Liwan District in southern China is 2.1%. Therefore, our study can better represent the mortality characteristics of the glaucoma population in China. It supports and extends these findings reporting an association between glaucoma and mortality, and it improves the understanding of glaucoma’s impact on the older population across China, one of the countries with the highest number of glaucoma cases [5,6].

In contrast to our study, the NHANES study showed no statistically significant association between glaucoma and mortality after adjusting for age and sex [15]. Several studies have found an association between glaucoma and mortality in univariate analyses, but the association disappeared after adjustment for confounders [12,30]. In general, the relationship between glaucoma and mortality varies considerably across different studies, but this may relate to factors such as race, geography, sampling quantity, and method. These factors may explain the differences in results between those studies and ours.

It is worth noting that our results clarify the association between glaucoma and all-cause mortality in different age groups by showing that, in people 75 years and older, age is an important component of increased all-cause mortality from glaucoma. This may be because aging is one of the main factors that promote the course of glaucoma and affect its treatment [31]. Few earlier studies in Asia have stratified their participants by age. The focus of some studies was the whole set of participants, without age stratification between younger and older groups, such as the study on this topic conducted in Taiwan [11]. Other studies focused on the association between glaucoma and mortality in people over 40 years, such as a rural cohort study from India and the above-mentioned study from Beijing [10,12]. As population aging continues worldwide, glaucoma among elderly people will exacerbate the disease and economic burden in Asia, especially in China. Aging will aggravate the disease’s severity and contribute to an increase in all-cause mortality. Our study is intended to raise more concerns about the survival status of glaucoma patients over the age of 75.

Potential mechanisms that may explain the increased risk of mortality in adults with glaucoma include adverse treatment effects and exposure to risk factors that are known to increase the risk of glaucoma and of major cause-specific deaths. First, adverse treatment effects from glaucoma surgery and medications are reported to be associated with high all-cause mortality rates. A retrospective cohort study from Korea indicated that all-cause mortality due to surgery for glaucoma was statistically significant (adjusted HR, 1.31; 95% CI, 1.05 to 1.62) for both open-angle glaucoma and angle-closure glaucoma [32]. The risk of death from neurological diseases was 2.7 times higher in older patients who underwent glaucoma surgery than in those who only received a diagnosis of glaucoma. Glaucoma medications can also cause severe side effects, including congestive heart failure (topical cholinergic agonists), increased blood pressure, and tachyarrhythmias (topical adrenergic agonists) [33]. The topical administration of a beta-blocker is one of the most common treatments for glaucoma; however, it is associated with increased mortality. Both a 2006 Australian study (relative risk [RR], 2.14; 95% CI, 1.18 to 3.89) and research conducted in the United States in 2008 (RR, 1.91, p = 0.04) showed higher mortality among glaucoma patients treated with topical timolol [26,34]. This could be attributed to the drug’s effect on blood lipid levels, such as a 20% reduction in high-density lipoprotein cholesterol and a 20-40% increase in triglycerides [35]. However, the conclusions were contradictory. A retrospective cohort analysis in the United States indicated that the use of any type of glaucoma medication had a statistically significant association with a 7% reduction in mortality (adjusted HR, 0.93; 95% CI, 0.90 to 0.95) [36]. Another United States study, from the same year, showed similar results (HR, 0.26; 95% CI, 0.16 to 0.40) [33]. The use of topical beta-blockers did not seem to be associated with excess mortality [37]. Second, exposure to risk factors known to increase the risk of both glaucoma and major cause-specific deaths (e.g., neurological disease, cardiovascular disease) may also contribute to high glaucoma-associated levels of mortality. The association between neurological diseases and glaucoma can be interpreted through the mechanisms that cause haemodynamic changes in the cerebral arteries and pathological substances, such as protein tau and amyloid-beta, which cause neurotoxicity and are also strongly associated with optic nerve damage in glaucoma [38]. It has been suggested that stroke [39- 41], Alzheimer’s disease [42-44], and Parkinson’s disease [45-47] are neurological diseases related to glaucoma. There was evidence that mortality in glaucoma patients was closely associated with cardiovascular events. A study in Australia indicated that cardiovascular mortality was 14.6% in patients with glaucoma as compared to 8.4% in non-glaucoma individuals, while further stratified analyses showed that cardiovascular mortality was higher among those previously diagnosed with glaucoma (RR, 1.85; 95% CI, 1.12 to 3.04) [26].

The current study has several strengths. First, it is a large-scale longitudinal study of elderly Chinese people with nationwide representation, a high participation rate, objective quality standards, and access to a complete, adjudicated registry of deaths. Second, the participants were randomly recruited from the general population; therefore, the results may be generalisable to the national elderly population. Last but not least, our study included an agestratified analysis, which enabled focusing on the elderly, especially those aged 75 years and older. This may help in evaluating the survival status of individuals with glaucoma in different age groups and provide a reference for future clinical decision-making.

Several study limitations should also be noted. First, the data on glaucoma were self-reported. Although there are some differences in evaluation between self-reports and clinical data, some studies support the reliability of self-reports for eye diseases including glaucoma [15,48,49]. Second, we did not collect information on the use of antiglaucoma medication, which might have explained the higher risk of mortality. Third, we explored the association between glaucoma and mortality, but did not include specific causes of death due to limited data. Further work could include the collection of medication information and participants’ specific causes of death to improve the accuracy of the analysis.

Despite these limitations, the present study suggests that glaucoma may be associated with a higher rate of mortality in middle-aged and elderly people in China, especially for those aged 75 years and older. This study provides an important reference for the design and evaluation of clinical glaucoma treatment and the management of patients of different ages.

## Figures and Tables

**Table 1.** Participant characteristics

Characteristics		Glaucoma		p-value
		Yes (n=141)	No (n=14,662)	
Age (yr)		66.6±10.1	59.0±9.9	<0.001
Sex				<0.001
	Male	47 (33.3)	7,039 (48.0)	
	Female	94 (66.7)	7,623 (52.0)	
Body mass index (kg/m^2^)		23.2±3.3	23.4±3.4	0.495
Education				0.013
	Primary or below	114 (80.9)	9,996 (68.2)	
	Middle school	16 (11.3)	3,014 (20.6)	
	High school	8 (5.7)	1,069 (7.3)	
	College or above	3 (2.1)	583 (4.0)	
Marital status				<0.001
	Married or partnered	110 (78.0)	12,869 (87.8)	
	Otherwise	31 (22.0)	1,793 (12.2)	
Smoking				0.036
	Yes	44 (31.2)	5,849 (39.9)	
	No	97 (68.8)	8,813 (60.1)	
Drinking				0.031
	None	109 (77.3)	9,801 (66.8)	
	Drink but less than once a month	8 (5.7)	1,138 (7.8)	
	Drink more than once a month	24 (17.0)	3,723 (25.4)	
Hypertension				<0.001
	Yes	52 (36.9)	3,519 (24.0)	
	No	89 (63.1)	11,143 (76.0)	
Dyslipidaemia				0.003
	Yes	24 (17.0)	1,315 (9.0)	
	No	117 (83.0)	13,347 (91.0)	
Diabetes				<0.001
	Yes	20 (14.2)	803 (5.5)	
	No	121 (85.8)	13,859 (94.5)	

Values are presented as mean±standard deviation or number (%).

**Table 2.** Cox proportional hazards regression models of all-cause mortality by glaucoma among all participants

Variables		HR (95% CI)	p-value
Glaucoma: Yes vs. no		1.46 (1.04, 2.03)	0.027
Age (yr)		1.09 (1.08, 1.09)	<0.001
Sex: Male vs. female		1.82 (1.59, 2.08)	<0.001
Body mass index (per 1 kg/m^2^)		0.92 (0.91, 0.94)	<0.001
Education: Primary or below vs. college or above		1.74 (1.28, 2.35)	<0.001
	Middle school vs. college or above	1.69 (1.22, 2.35)	0.002
	High school vs. college or above	1.24 (0.81, 1.89)	0.317
Marital status: Married or partnered vs. other		0.39 (0.35, 0.44)	<0.001
Smoking: Yes vs. no		1.25 (1.09, 1.42)	0.001
Drinking: Drink less than once a month vs. drink more than once a month		0.84 (0.67, 1.07)	0.164
	None vs. drink more than once a month	1.38 (1.22, 1.56)	0.001
Hypertension: Yes vs. no		1.80 (1.62, 2.01)	<0.001
Dyslipidaemia: Yes vs. no		0.88 (0.73, 1.06)	0.320
Diabetes: Yes vs. no		1.70 (1.42, 2.03)	<0.001

HR, hazard ratio; CI, confidence interval.

**Table 3.** Cox proportional hazards regression models of all-cause mortality by glaucoma per age group

Variables		Participants aged (yr)			
		45-74 (n=13,572)	p-value	≥75 (n=1,231)	p-value
Glaucoma: Yes vs. no		1.39 (0.81, 2.41)	0.235	1.89 (1.24, 2.89)	0.003
Sex: Male vs. female		1.99 (1.67, 2.36)	<0.001	1.21 (0.97, 1.50)	0.088
Body mass index (per 1 kg/m^2^)		0.93 (0.92, 0.95)	<0.001	0.98 (0.95, 1.00)	0.062
Education: Primary or below vs. college or above		1.69 (1.18, 2.42)	0.004	1.86 (1.04, 3.32)	0.037
	Middle school vs. college or above	1.30 (0.89, 1.89)	0.172	0.73 (0.33, 1.60)	0.432
	High school vs. college or above	0.71 (0.45, 1.13)	0.152	2.14 (0.69, 6.67)	0.188
Marital status: Married or partnered vs. otherwise		0.53 (0.45, 0.62)	<0.001	0.83 (0.69, 0.99)	0.038
Smoking: Yes vs. no		1.34 (1.14, 1.58)	<0.001	1.26 (1.03, 1.55)	0.027
Drinking: Drink less than once a month vs. drink more than once a month		0.76 (0.58, 1.01)	0.055	1.00 (0.63, 1.57)	0.993
	None vs. drink more than once a month	1.34 (1.15, 1.55)	<0.001	1.33 (1.06, 1.68)	0.015
Hypertension: Yes vs. no		1.78 (1.55, 2.04)	<0.001	1.25 (1.04, 1.49)	0.015
Dyslipidaemia: Yes vs. no		0.88 (0.71, 1.09)	0.245	0.95 (0.68, 1.33)	0.784
Diabetes: Yes vs. no		2.03 (1.65, 2.50)	<0.001	1.03 (0.72, 1.49)	0.860

Values are presented as hazard ratio (95% confidence interval).

## SUPPLEMENTARY MATERIALS

Supplementary Material 1. Baseline characteristics by glaucoma in those who died among all population

Supplementary Material 2 Baseline characteristics by glaucoma in those who died in different age group
