# Supplementary material for: The association between glaucoma and all-cause mortality in middle-aged and elderly Chinese people: results from the China Health and Retirement Longitudinal Study
Source: Epidemiol Health. 2023 Jul 21;45:e2023066. doi: 10.4178/epih.e2023066 (PMC10667574; doi:10.4178/epih.e2023066)
Supplement: Supplementary Material 1. — Baseline characteristics by glaucoma in those who died among all population [file epih-45-e2023066-Supplementary-1.docx]

**Supplementary Material 1. Baseline characteristics by glaucoma in those who died among all population**

| **Variables** | **Glaucoma** | | ***P*-value** |
| --- | --- | --- | --- |
|  | **Yes (n=36)** | **No (n=1591)** |  |
| **Mean age (yrs)** | 75.94 (8.078) | 68.99 (10.887) | **<0.001** |
| **Gender** |  |  | **0.016** |
| Male | 38.9% (14) | 58.9% (937) |  |
| Female | 61.1% (22) | 41.1% (654) |  |
| **BMI** | 22.5 (3.2) | 22.4 (3.3) | 0.990 |
| **Education** |  |  | 0.064 |
| Primary or below | 94.4% (34) | 81.0% (1288) |  |
| Middle school | 2.8% (1) | 13.6% (216) |  |
| High school | 2.8% (1) | 2.7% (43) |  |
| College or above | 0.0% (0) | 2.8% (44) |  |
| **Marital status** |  |  | 0.137 |
| Married or partnered | 61.1% (22) | 72.3% (1151) |  |
| Otherwise | 38.9% (14) | 27.7% (440) |  |
| **Smoking** |  |  | **0.015** |
| Yes | 30.6% (11) | 51.0% (811) |  |
| No | 69.4% (25) | 49.0% (780) |  |
| **Drinking** |  |  | 0.102 |
| None | 86.1% (31) | 69.6% (1108) |  |
| Drink but less than once a month | 2.8% (1) | 5.2% (82) |  |
| Drink more than once a month | 11.1% (4) | 25.2% (401) |  |
| **Hypertension** |  |  | 0.585 |
| Yes | 30.6% (11) | 34.9% (556) |  |
| No | 69.4% (25) | 65.1% (1035) |  |
| **Dyslipidaemia** |  |  | 0.396 |
| Yes | 11.1% (4) | 8.8% (140) |  |
| No | 88.9% (32) | 91.2% (1451) |  |
| **Diabetes** |  |  | 0.297 |
| Yes | 13.9% (5) | 8.9% (141) |  |
| No | 86.1% (31) | 91.1% (1450) |  |

Data are presented as % (N) or mean (standard deviation). Comparisons were for the glaucoma group with the No glaucoma group.
