# Supplementary material for: The association between glaucoma and all-cause mortality in middle-aged and elderly Chinese people: results from the China Health and Retirement Longitudinal Study
Source: Epidemiol Health. 2023 Jul 21;45:e2023066. doi: 10.4178/epih.e2023066 (PMC10667574; doi:10.4178/epih.e2023066)
Supplement: Supplementary Material 2 — Baseline characteristics by glaucoma in those who died in different age group [file epih-45-e2023066-Supplementary-2.docx]

**Supplementary Material 2. Baseline characteristics by glaucoma in those who died in different age group**

| **Variables** | **45 to 74 Years of Age (n=1064)** | | | **75+ Years of Age (n=563)** | | | |
| --- | --- | --- | --- | --- | --- | --- | --- |
|  | **With**  **glaucoma (n=13)** | **Without glaucoma (n=1051)** | ***P*-value** | **With**  **glaucoma**  **(n=23)** | **Without glaucoma (n=540)** | | ***P*-value** |
| **Mean age (yrs)** | 67.4 (6.7) | 62.9 (7.8) | **0.041** | 80.8 (3.4) | | 80.7 (4.6) | 0.979 |
| **Gender** |  |  | 0.443 |  | |  | 0.086 |
| Male | 53.8% (7) | 64.1% (674) |  | 30.4% (7) | | 48.7% (263) |  |
| Female | 46.2% (6) | 35.9% (377) |  | 69.6% (16) | | 51.3% (277) |  |
| **BMI** | 23.6 (3.7) | 22.8 (3.3) | 0.418 | 21.8 (2.8) | | 21.7 (3.1) | 0.905 |
| **Education** |  |  | 0.080 |  | |  | 0.774 |
| Primary or below | 92.3% (12) | 73.8% (776) |  | 95.7% (22) | | 94.8% (512) |  |
| Middle school | 0(0) | 19.4% (204) |  | 4.3% (1) | | 2.2% (12) |  |
| High school | 7.7% (1) | 3.7% (39) |  | 0(0) | | 0.7% (4) |  |
| College or above | 0(0) | 3.0% (32) |  | 0(0) | | 2.2% (12) |  |
| **Marital status** |  |  | 0.361 |  | |  | 0.411 |
| Married or partnered | 92.3% (12) | 82.7% (869) |  | 43.5% (10) | | 52.2% (282) |  |
| Otherwise | 7.7% (1) | 17.3% (182) |  | 56.5% (13) | | 47.8% (258) |  |
| **Smoking** |  |  | 0.223 |  | |  | 0.120 |
| Yes | 38.5% (5) | 55.4% (582) |  | 26.1% (6) | | 42.4% (229) |  |
| No | 61.5% (8) | 44.6% (469) |  | 73.9% (17) | | 57.6% (311) |  |
| **Drinking** |  |  | 0.542 |  | |  | 0.130 |
| None | 76.9% (10) | 66.0% (694) |  | 91.3% (21) | | 76.7% (414) |  |
| Drink but less than once a month | 7.7% (1) | 5.6% (59) |  | 0 (0) | | 4.3% (23) |  |
| Drink more than once a month | 15.4% (2) | 28.4% (298) |  | 8.7% (2) | | 19.1% (103) |  |
| **Hypertension** |  |  | 0.824 |  | |  | 0.498 |
| Yes | 30.8% (4) | 33.7% (354) |  | 30.4% (7) | | 37.4% (202) |  |
| No | 69.2% (9) | 66.3% (697) |  | 69.6% (16) | | 62.6% (338) |  |
| **Dyslipidaemia** |  |  | 0.484 |  | |  | 0.790 |
| Yes | 15.4% (2) | 9.6% (101) |  | 8.7% (2) | | 7.2% (39) |  |
| No | 84.6% (11) | 90.4% (950) |  | 91.3% (21) | | 92.8% (501) |  |
| **Diabetes** |  |  | 0.142 |  | |  | 0.555 |
| Yes | 23.1% (3) | 10.5% (110) |  | 8.7% (2) | | 5.7% (31) |  |
| No | 76.9% (10) | 89.5% (941) |  | 91.3% (21) | | 94.3% (509) |  |

Data are presented as % (N) or mean (standard deviation). Comparisons were for the glaucoma group with the No glaucoma group.
